# Perceptions and needs of patients, caregivers and health professionals regarding an oncology community center: a qualitative study

**DOI:** 10.3389/fpubh.2025.1569994

**Published:** 2025-06-27

**Authors:** Inbal Mitnik, Ilit Turgeman, Svetlana Baziliansky, Hassan Shalabna, Leena Nassar-Sakas, Gil Bar-Sela

**Affiliations:** ^1^Division of Oncology, Rambam Health Care Campus, Haifa, Israel; ^2^The Oncology Service, Lin Medical Center, Clalit Health Services, Haifa, Israel; ^3^The Oncology Department, Carmel Medical Center, Haifa, Israel; ^4^School of Social Work, Faculty of Social Welfare and Health Sciences, University of Haifa, Haifa, Israel; ^5^Cancer Center, Emek Medical Center, Afula, Israel; ^6^Bruce Rappaport Faculty of Medicine, Technion-Israel Institute of Technology, Haifa, Israel

**Keywords:** cancer care, caregivers, community care, patients, oncology care

## Abstract

**Introduction and aims:**

Oncology community centers (OCCs) may be beneficial for patients residing in regions requiring prolonged travel to large hospitals. A new model of OCC has been established in a peripheral region of Northern Israel, in affiliation with a large hospital. This center aims to increase accessibility to medical care and to make it more patient centered. The current study aims to better understand the needs and perceptions of patients, caregivers, and health professionals regarding the OCC to integrate them into the newmodel OCC and adapt the medical services to the population’s needs.

**Materials and methods:**

Semi-structured interviews were conducted with 18 participants to explore their perceptions regarding the new OCC. The interviews were qualitatively analyzed using the interpretative phenomenological analysis method.

**Results:**

Patients and caregivers recognized the advantages of receiving treatment closer to home but were concerned about losing their sense of security. Nurses expressed the need to increase their confidence by communicating closely with the hospital’s staff and ensuring safety in case of urgent situations. Physicians emphasized the importance of hospital and community collaboration and the potential to enhance treatment adherence.

**Conclusion:**

The results indicate that the new OCC was perceived as a significant step in developing medical services, however the main concern was a decrease in the level of confidence due to its distance from the hospital. Strong collaboration between academic and community settings is essential to ensure continuity of care and a sense of security.

## Introduction

Cancer treatment programs worldwide comprise various institutes and centers that differ in size, services provided, and academic affiliations (1). These range from academic comprehensive cancer institutes, hospital-affiliated centers with limited services, and community-based programs (1). In Israel, cancer care is provided in cancer centers located within 17 hospitals (Seven of them in main cities and 10 in peripherical locations), all with an academic affiliation (The ministry of health). These cancer centers provide comprehensive cancer care and psychosocial services, with different types of oncological treatment, and including surgery, radiation therapy and chemotherapy. However, for individuals with cancer living in peripheral areas, prolonged travel to the nearest hospital constitutes a significant burden. Facilitating access to cancer care in the community may play a significant role in improving patients’ quality of life and even cancer-related outcomes.

Individuals with cancer often experience cancer and treatment-related symptoms, ranging from pain, fatigue and nausea to psychological, cognitive, and neurological impairments and even life-threatening complications (2). Patients often require the assistance of caregivers, which may lead to emotional and financial burden (3–5). Due to significant developments in cancer care and enhanced patient survival, extended treatment schedules may add additional challenges for patients and their caregivers (6–8). A compromised physical state may preclude patients from traveling to large centers far from home (9).

The health care system in Israel, is based on the National Health Insurance Law of 1994, which states that every Israeli resident is entitled to health insurance coverage through one of four nonprofit health maintenance organizations (HMOs), including full coverage for cancer treatment (10). The HMOs must provide all insured people with standard and equal health services, as specified by law (10). However, according to the Israeli Ministry of Health (11), there are discrepancies in health parameters that may be related to education, income, and geographical location. Although the peripheral regions consist of communities with various socioeconomic levels (12), they feature higher rates of morbidity and a lower ratio of physicians, nurses, and hospital beds per person compared with central areas (11).

Therefore, recently, one of the main HMOs decided to establish an oncology community center (OCC) in the northern district of Israel. The center provides oncological treatments such as chemotherapy, immunotherapy, and biological therapy. The staff at the center include doctors from a large oncology center, as well as nurses, a nutritionist, a social worker and a secretary, that worked in community settings and have received specific oncology training. The center’s daily capacity is up to 10 patients. A community center that serves as a branch of a hospital and includes medical staff from the hospital is unique in Israel. This new OCC concept is similar to hospital-associated cancer programs in the United States (7). It aims to increase the accessibility of medical services in the community and patient adherence to treatment, improve quality of life and health outcomes, while reducing health discrepancies. Given the lack of data regarding the OCC model in Israel and the well-known difficulty to acclimate to new services (13), it is highly important to understand the perceptions and needs of patients, caregivers, and health care professionals (13). This may assist in adapting medical services to the population’s needs, decreasing discrepancies due to health parameters, improving quality of care, and increasing patient satisfaction. In addition, this knowledge may assist in developing OCCs in other parts of the country. The aim of the present study was to examine the perceptions of patients, family members, physicians (oncologists and general practitioners [GPs]), and nurses of the proposed OCC, including their needs, desires, and concerns regarding the OCC.

## Materials and methods

### Participants and procedure

The study was carried out in Emek Medical Center, a large hospital in northern Israel. To obtain a variety of viewpoints and thoughts regarding the research topic, the study team recruited participants using convenience sampling. The sample included five patients who were scheduled to receive treatment in the new OCC, three family members, three oncologists, five oncology nurses, and two GPs. Due to practical constraints, it was not possible to have a larger and more diverse sample. However, the sample was diverse in demographic characteristics and interviews generally showed data saturation, meaning it was established that additional interviews will not add any value to the findings.

This study adopted a focused approach centered on OCC, in contrast to broader qualitative investigations of complex experiences and perceptions. The sample—comprising patients, family members, and professionals—was clearly delineated, and the high quality of the dialogue yielded rich, comprehensive data that effectively addressed the research questions.

Patients and caregivers learned about the study during their routine visits to a physician. Only one patient refused to participate in the study. Health professionals were informed during routine staff meetings or by electronic mail. Individuals who were interested in participating in the study met the main researcher at the hospital and were informed about the study objectives and procedure. They signed an informed consent form. The study was approved by the ethics board of the hospital (#0140-22-EMC).

### Data collection

Semi structured interviews were conducted with patients, caregivers, and health professionals based on an interview guide. The interview guide was developed according to the service implementation (14), to reflect the various aspects concerning the new OCC. All interviews were conducted by a senior social worker, with extensive experience with this technique and with no affiliation with any medical center. The interviews occurred in a private room at the hospital. They lasted 40–50 min. The goal of a semi structured interview is to create an open dialogue between the interviewer and interviewee and provide the opportunity for people to tell their story openly and freely with minimal interference (15).

At the interview, participants were informed about the new OCC that was recently opened with a short description, its aims, and functioning procedures. Sample questions of the interview guide are: “What do you think about the center?” “In your opinion, what are the advantages and disadvantages of the center?” “In your opinion, what are the essential aspects that should be included in the center?” “Do you have any concerns regarding the center?”

During the interviews, a recording device was used to document the interviews as precisely as possible. The interviews were transcribed while ensuring that personal information remained confidential.

### Data analysis

The interviews from each group (patients, caregivers, and health professionals) were analyzed using the interpretative phenomenological analysis method (15). This method aims to investigate people’s experiences from their subjective point of view, emphasizing how they make sense of their personal and social world. This method involved several phases. First, each transcript was read closely several times; significant topics were marked and attention was directed to language and use of key words or metaphors. In the next phase, the main topics of each interview were identified and conceptualized into themes, or concise phrases that aim to capture the essence of the text. Analytic and theoretical connections between themes were identified to compose superordinate themes (15). The analysis was conducted by a senior psychologist with the support of another researcher, both with extensive experience with the method. In addition, two researchers reviewed the interviews to cross-check the data and to ensure the conclusions that emerged from it.

## Results

Eighteen participants were included in the study. Their demographic characteristics are detailed in Table 1. Two themes were identified. The themes and subthemes that emerged from the interviews are detailed here.

**Table 1:** Demographic characteristics of participants.^a^

Group	Age	Gender	Years of education	Religion	Type of cancer	Descriptor
Patient	76–80	Male	12	Jewish	Melanoma	Patient
	51–55	Female	12	Jewish	Breast cancer	Patient
	56–60	Female	17	Jewish	Breast cancer	Patient
	41–45	Female	15	Jewish	Breast cancer	Patient
	81–85	Female	15	Jewish	Carcinoma	Patient
Caregiver	71–75	Female	12	Jewish		Wife
	41–45	Male	14.5	Jewish		Husband
	81–85	Male	15	Jewish		Husband
Health professional	36–40	Male	19	Muslim		Oncologist, OCC (planned)
	56–60	Male	19	Jewish		Oncologist, hospital
	41–45	Female	19	Jewish		Nurse, OCC (planned)
	36–40	Female	15	Muslim		Nurse, OCC (planned)
	31–35	Female	15	Muslim		Nurse, OCC (planned)
	41–45	Female	20	Jewish		Nurse, OCC (planned)
	36–40	Female	19	Jewish		Oncologist, hospital
	41–45	Female	19	Muslim		Nurse, hospital
	51–55	Male	27	Jewish		GP
	41–45	Female	20	Jewish		GP

a All participants were married.

*Theme 1: “You Just Need Someone Who Smiles at You”: Perceptions of the New Center and Its Essential Features.*

### Sense of security

All participants mentioned that for patients to choose to receive their treatment at the OCC, it is needed to provide a sense of security. According to patients, achieving trust and confidence in the health care team could be achieved in several ways. First, they mentioned the need to provide a professional and experienced team: “The most important thing is to bring professional and experienced staff, not new people. … It will make you feel secure and confident” (Patient 8).

Additionally, patients described the need for close and continuous connection with the hospital and its experts. “Just do copy paste from [name of hospital]. … They are very experienced. … If they will communicate with the new center … and Professor [name of professor from the hospital] can come here once a month” (Patient 5).

Caregivers also mentioned the need to be reassured that the new center would be affiliated with the hospital, which was perceived as professional: “The staff in [name of large hospital] is great, so if the nurses will come from there, it will be great and very reassuring” (Caregiver 12).

### Professional collaboration

Health professionals, especially nurses who will staff the center, also mentioned the need to be affiliated with the hospital to increase the sense of confidence in the staff and among patients. “[Collaboration with the hospital] should be very close. … The physicians are coming from there” (Nurse 7). However, this concern related to a broader issue: the need for professional collaboration, given the complexity of cancer care: “Cancer care is very complicated—there is complementary medicine, the social worker, the dietitian. It is not just the nurses and the chemotherapy. … We need to have more staff members. … It should be more holistic” (Nurse 7). In addition, they emphasized the importance of collaboration with GPs in the community. They mentioned that this would ensure continuity of care and increase patients’ sense of security, as one oncologist described:

“The collaboration with the GP is one of the most important aspects. … The oncological treatments can be very intensive. … If the GP is involved., … it’s more reassuring. Someone takes care of you in the hospital and in the community as well. We work together” (Oncologist 15).

A GP described a similar approach:

“Ideally, I would give them [oncologists in the OCC] my phone number and they would give me theirs … to have direct communication, rather than sending a referral and waiting for a reply. … It can make it easier for the patients” (GP 18).

Health professionals who planned to work in the new OCC also referred to the importance of collaboration with GPs from a strategic point of view. They mentioned the need to publicize the new center and ensure referrals of patients by GPs in the community: “The most important thing is to establish the connection, to spread the news regarding the new center, … to inform them about the new place so they can refer patients to us” (Oncologist 3).

Cancer patients and caregivers perceived the oncology team as the focus of care, whereas their GP was perceived as less related to cancer care, as one patient shared: “I had no connection with my GP. … I did not think he could help me. … The oncologist provided all the answers. … I did not feel as if I needed something from the GP … rather than specific questions or Covid-19 vaccinations” (Patient 6).

### Interpersonal environment

Patients and caregivers referred to the interpersonal environment as another feature of the new OCC. For such a center to be attractive, they emphasized the need for a supportive attitude from the staff to establish a warm and pleasant atmosphere for patients and reassure them, as one patient described: “Support is one of the most important things. You need to come here not just for the treatment, but for the support” (Patient 8). They expressed their belief that a caring staff would provide a homely and caring environment: “Warm and kind staff with a smile. … They [patients] should not be nervous even if they are having a bad day” (Caregiver 1). Health care professionals supported the importance of this aspect, as described by a nurse: “To give a homely feeling. … The patient should feel comfortable to come here. … The dynamic between staff members should be pleasant, positive energies” (Nurse 7).

Patients also emphasized the need for the staff to be available, so they do not have to wait in line or for a long time for their appointment. This may be related to the difficulty they had experienced in long travel to hospitals and in waiting for test results and medical procedures: “It is important that there will be a specialist at any given moment. … The nurses must be available. It is very, very important that they give patients their phone number” (Patient 8).

These aspects were perceived by patients as more important than the physical environment, as one patient shared: “The curtains there [in the hospital] were torn, the couch, the floor was old, but who cares? … You just need someone who smiles at you” (Patient 5). Mostly health professionals mentioned the importance of the physical environment of the new OCC: “The building should be designed like any other medical facility: … accessible and in a convenient location. … Parking space is very important, especially for cancer patients” (Oncologist 3), “More privacy, curtains, to respect the patients. … They should have a coffee and tea corner, something to eat. … They come from the morning till the evening” (Nurse 10).

*Theme 2: “In the Hospital, You Have a Complete Staff and You Feel Safer”: Pros and Cons of the Proposed Community Center.*

### Closer location to home

Patients said they recognized the main advantage of the new center as being closer to their home, which may shorten their journey for treatment and make receiving care easier for them. For example, “It is more accessible, and you do not need to go through the whole process each time. … I just leave the house, and I do not need to worry about traveling for 3.5 h” (Patient 8), “There will not be all the traveling, all the hassle, even your caregiver will not have to be with you the whole day” (Patient 6).

Similarly, the caregivers stressed that the proximity of treatment facilities could make treatments easier for patients. “It will be excellent for the patients going through these difficult treatments. They will not have to travel too much. … It will be much easier for them” (Caregiver 9).

Health professionals also referred to the advantages of a closer location, stressing its relevance for improving patients’ well-being: “It will be easier for them. … They suffer so much. … At least they will have the treatments nearby” (Nurse 13). Like the patients, they also mentioned the advantage of proximity for caregivers: “One needs to take a day off from work to go with a sick parent to the hospital, so obviously this is an improvement” (GP 18). They also stated that it may increase caregivers’ involvement in care: “It will help their family to be with them. They will be more involved” (Nurse 16).

Another advantage for patient care identified by health care professionals was that proximity to care facilities may increase efficiency due to more timely provision of treatment: “In oncology, timing is highly important. … Sometimes because of the distance, patients aren’t able to get the treatment on time. … The center can improve the efficacy of the oncological treatment” (Oncologist 15). An additional advantage presented by health care professionals was that proximity to treatment may increase adherence, because the journey might not be so exhausting. “It is highly important because the patient does not have to think twice whether to come and have the treatment. It will improve their adherence” (Nurse 16).

Finally, physicians referred to the location of the OCC from a broader and strategic perspective, including the contribution to advancing of medical services in peripheral regions of Israel: “It is a great idea. … For many years, the population in the peripheral areas had no adequate health services and they had to travel for miles for medical care” (Oncologist 15).

### Decreased level of confidence

Regarding the disadvantages of the new center, patients and caregivers mentioned that being away from the hospital, which was perceived as large and experienced, may decrease their level of confidence. “You have one physician, while in the hospital, you have a complete staff, and you feel safer” (Patient 8). This concern was perceived to be extra challenging for patients who started treatment in the hospital and had already established trust with their oncologist: “We will not leave our professor [in the hospital]” (Caregiver 1).

Health care professionals mirrored these patient concerns: “The main disadvantage is the patients’ confidence. … There [in the hospital] they are treated by a large and professional staff. … Here they may be worried” (Oncologist 4). Their concerns related to the staff’s experience as well: “The first time is always scary, for them and for us as nurses without experience. Obviously, it will be difficult in the beginning” (Nurse 10), “We are starting from zero. It’s a bit stressful. You cannot help wondering whether it will work” (Oncologist 3). Health care professionals pointed out that distance may be a problem, particularly in case of emergency: “Being away from the hospital, especially during an emergency, may challenge the sense of security of patients and staff” (Nurse 13), “If something happens, we need to evacuate the patient by an ambulance. … It’s a 40-min drive to the nearest hospital” (Nurse 7). These concerns also emerged in the perceptions of health professionals regarding the type of patients and treatments suitable for the new center, which included stable patients with simple protocols to minimize the possibility of medical complications: “We need to choose the patients carefully. … Some treatments are more complicated, … they require more skill … [and] more staff” (Oncologist 15).

Health professionals referred to the perception that the advantages of the new center may also be perceived as disadvantages for both patients and staff members. On one hand, it could be a homely and nearby center. On the other hand, it may be less secure and less familiar, as demonstrated by a nurse who planned to work in the OCC:

“It is nice to get to a new, quiet, and clean place; however, there might be some concerns because it is new. … They might think, “Am I the first patient here? Why am I the only patient here?” … In the community, you [staff member] are by yourself. You need to know everything because if God forbid something happens, the drive [to the hospital] is at least 40 min. … On the other hand, it is nice to get to a community setting. You do not feel [the same] in a hospital, which is perceived as more intimidating” (Nurse 14).

This perception may also reflect the concerns of the staff, particularly those who planned to work in the new OCC, regarding their responsibility and sense of confidence.

## Discussion

The current study qualitatively explored the needs and perceptions regarding establishing an OCC from the viewpoints of patients, caregivers, and health professionals. These findings provide several key implications for the health system in Israel and other countries that are changing the delivery of cancer care and other health services for populations in peripheral regions of the country, to improve quality and accessibility.

In the case of the proposed OCC, aspects such as stability and familiarity were perceived as more significant than the physical environment or travel time to the hospital that were noted by staff. This may reflect patients’ emotional experience, which includes dealing with existential uncertainty, changes in close relationships, and a sense of loss of control (16). This may increase the difficulty of dealing with change and emphasize the need for stability. Addressing these emotional aspects of patients, particularly those moving from the hospital, should be considered a main goal for the new OCC and its vision. It should create a homely and pleasant environment for patients, and the staff should be available and supportive. This may address patients’ need for security and support.

Patients and caregivers perceived the advantages of receiving treatment in a medical center closer to home. However, they were worried about losing their sense of security and familiarity in a new place with a new staff far away from the hospital. The participating nurses, especially those planned to work at the OCC, were concerned about possible emergencies or the need to make urgent decisions. They felt they would have greater responsibility compared to being a part of a large team at a hospital. Patients and healthcare professionals also stressed the need for collaboration with a large hospital to ease their concerns, particularly during the initial stages of establishing the OCC. This may increase their sense of security and familiarity by enabling continuity of care.

Health professionals emphasized the importance of the GPs’ involvement in patient care to establish a more integrated model of care. They believed that the geographical location of the new center inside the community would promote mutual and professional collaboration with community health services, especially with GPs… and they perceived this as an important aspect of ensuring continuity of care and facilitating patients’ sense of security. The need for collaboration between academic and community settings has been described in previous studies. This partnership was described as a key component in the model of community health centers that could facilitate high-quality care for patients (17, 18). Another study demonstrated that an academic—community partnership enabled the efficient establishment of a screening intervention protocol according to updated health recommendations (19).

In this study, patients were less keen about the role of their GPs in their cancer care. A previous study demonstrated that patients felt a strong bond with their oncologist and were reluctant to give up this trusted relationship (20). They expected that this health professional would be involved in their cancer care and felt more reassured by their presence. Patients often perceive GPs as generalists, more suited to the management of comorbidities and preventive care and lacking the adequate training or knowledge to be involved in cancer care. This perception was demonstrated among GPs as well, who reported a sense of not having adequate training in cancer care and being disconnected from the process (20). Participating oncologists said that it was possible for GPs to be more involved in cancer care if they had a clearly predefined role (20). This model of integrated and shared care can provide continuity of care for cancer patients, considering their psychosocial needs and management of comorbidities (21). It may reassure patients, take the burden off the hospital system, and increase GPs’ involvement (20, 21). An essential part of shared cancer care is multidisciplinary teamwork, with effective and timely communication between primary and secondary care providers (20). This aspect was demonstrated in the current study, with health professionals suggesting that information sharing, availability, and direct communication with GPs is necessary to improve medical collaboration. Given the importance placed on collaboration by health professionals, this issue should be further investigated to strengthen patients’ understanding regarding the role of GPs in their cancer care.

The current study examined a unique model of an OCC affiliated with a hospital in Israel. However, this approach may be relevant to similar medical centers in the United States and worldwide, such as hospital associated cancer programs (7). In particular, the results indicate that perceptions, concerns, and needs may differ between patients and staff and among different health professional groups. Previous research has demonstrated that the assimilation of a new health service is usually accompanied by concerns among patients and healthcare providers alike (13). Staff members of different professions often have varying perceptions and attitudes toward changes in the service setting. Therefore, it is essential to thoroughly explore their perceptions and needs to ensure the successful implementation of a new service (13).

The current study has several limitations. It featured a relatively small sample, so the ability to extrapolate findings to other populations is limited. The patients’ sample was not diverse enough in terms of ethnicity and religion, which may also limit the ability to extrapolate findings. In addition, we used qualitative measures only, which may limit our understanding regarding needs and perceptions of the new center.

To conclude, the current qualitative study demonstrated that the main needs regarding the new OCC were sense of security, professional collaboration and interpersonal environment. The model was perceived as a significant step in developing medical services, but the main concern was a decrease in the level of confidence due to its distance from the hospital. Key goals include defining and strengthening collaboration between the academic setting, GPs, and the community to ensure continuity of care and a sense of security and familiarity for patients and caregivers alike. Follow-up interviews for all the participants are required to evaluate their perceptions after the OCC has established and operated for an extended period. This may assist to better evaluate the new model. Future studies should include larger samples with quantitative measures to better evaluate the needs of the population regarding this new cancer care model as well as the potential for enhanced health outcomes.

## Data Availability

The raw data supporting the conclusions of this article will be made available by the authors, without undue reservation.
